# Titanium Implants Functionalized with Zoledronic Acid Associated with Ruterpy Accelerate Peri-Implant Repair in Healthy and Osteoporotic Rats

**DOI:** 10.3390/biomimetics10080547

**Published:** 2025-08-20

**Authors:** Laura Vidoto Paludetto, Isadora Breseghello, Sabrina Cruz Tfaile Frasnelli, Fábio Roberto de Souza Batista, Paulo Roberto Botacin, Cristina Antoniali, Paulo Noronha Lisboa-Filho, Roberta Okamoto

**Affiliations:** 1 Department of Basic Sciences, Araçatuba Dental School, São Paulo State University Júlio de Mesquita Filho—UNESP, Aracatuba 16015-050, SP, Brazil; isadora.breseghello@unesp.br (I.B.); satfaile@yahoo.com.br (S.C.T.F.); fabio.rs.batista@unesp.br (F.R.d.S.B.); paulo.botacin@unesp.br (P.R.B.); cristina.antoniali@unesp.br (C.A.); 2 Department of Physics and Meteorology, Bauru Sciences School, São Paulo State University Júlio de Mesquita Filho—UNESP, Bauru 17033-360, SP, Brazil; paulo.lisboa@unesp.br

**Keywords:** bone regeneration, osseointegration, zoledronic acid, osteoporosis, ovariectomy, rats, wistar, confocal microscopy, tomography, X-ray computed, dental implants, surface properties

## Abstract

Osteoporosis compromises bone quality and impairs implant osseointegration. Since an adequate bone bed is essential for implant stability and success, this study evaluated the effects of implant surface functionalization with zoledronic acid (ZOL), alone or combined with ruterpy (TERPY), on peri-implant bone healing in healthy (SHAM) and osteoporotic (OVX) rats. ZOL has antiresorptive properties, while TERPY exhibits osteoinductive potential. The hypothesis was that ZOL + TERPY would act synergistically by inhibiting bone resorption and promoting new bone formation. Sixty-six female Wistar rats (3 months old) were divided into six groups (*n* = 11) according to systemic condition (SHAM or OVX) and implant type: conventional (CONV), ZOL, or ZOL + TERPY. Surgeries (sham or bilateral ovariectomy) were performed on day 0, and implants were placed in the tibial metaphysis on day 90. Fluorochromes were administered on days 104 (calcein) and 114 (alizarin), and euthanasia was performed on day 118. Samples were analyzed histologically via confocal microscopy and micro-computed tomography (Micro-CT). The ZOL + TERPY groups demonstrated significantly accelerated peri-implant bone repair, showing greater bone formation and organization; improved BV/TV, Tb.N, and I.S.; and reduced Tb.Sp and Po.Tot compared to CONV and ZOL-alone groups. In conclusion, ZOL + TERPY enhances and speeds bone healing, even under osteoporotic conditions.

## 1. Introduction

Over the past decades, the global demographic transition has been marked by population aging as a result of biomedical advances, improved healthcare coverage, and broader access to public health resources. This phenomenon, especially evident in industrialized and developing countries, has been associated with optimistic projections regarding the future longevity of their populations [1].

However, the prolongation of life also increases the incidence of non-communicable chronic diseases, among which osteometabolic disorders stand out. Osteoporosis is prominent in this group due to its prevalence and clinical impact. This condition is characterized by an imbalance between bone formation and resorption processes, leading to reduced mineral density, weakening of the bone matrix, and increased fracture risk [2]. The hormonal decline that accompanies aging—such as the abrupt drop in estrogen levels during menopause—compromises the regulatory mechanisms of osteoclastic and osteoblastic activity, disrupting the balance required to maintain bone tissue [3,4].

This deterioration in bone quality has direct implications for restorative surgical procedures, such as dental implants, which have been widely used for functional and aesthetic oral rehabilitation in edentulous patients. The literature reports high clinical success rates and predictability [5,6,7]. However, the effectiveness of the osseointegration process—defined as the direct contact between viable bone and the implant surface under functional load [8]—can be compromised in individuals with low bone density. Several studies suggest that osteoporotic bone resembles type IV bone, characterized by low trabecular density and a thin cortical layer, which negatively affects the initial stability of implants [9]. Although systematic reviews have not identified a formal contraindication for implant placement in osteoporotic patients [10,11], contradictory results still persist in the literature, with reports of increased marginal bone loss [12,13], higher failure rates [14], and reduced extracellular matrix expression in animal models [9].

The most widely used pharmacological treatment for osteoporosis involves systemic bisphosphonates, such as zoledronic acid, which act by inhibiting osteoclastic activity and reducing bone resorption [15]. Despite clinical benefits in controlling bone loss and preventing fractures, these drugs may trigger significant adverse effects, including gastrointestinal disturbances, renal dysfunction, immune reactions, ocular events, and the risk of osteonecrosis of the jaw [16]. These side effects have motivated the search for alternative therapeutic strategies, focusing on less invasive and more targeted local approaches.

In this context, surface modification of implants with locally acting biomolecules emerges as a promising alternative. The dip-coating technique allows for the incorporation of bioactive agents into the implant surface, enabling controlled and localized drug release directly into the peri-implant bone bed. This approach aims to stimulate a favorable cellular response with the potential to counteract damage caused by systemic conditions such as osteoporosis.

Among these agents, zoledronic acid—a potent nitrogen-containing bisphosphonate with targeted action on bone tissue [17,18]—has shown positive results in studies utilizing its local application on implantable surfaces, both in healthy animals and estrogen deficiency models [19,20,21,22,23,24,25,26]. Another notable compound is ruterpy ([Ru(terpy)(bdq)NO]^3+^), a ruthenium complex synthesized at the School of Pharmaceutical Sciences of Ribeirão Preto (FCFRP-USP), which acts as a nitric oxide donor. Nitric oxide plays a key role in bone regeneration due to its ability to promote vasodilation, induce angiogenesis, and stimulate the production of factors such as VEGF, in addition to modulating cellular activity involved in bone remodeling. Previous studies conducted by our group with ruterpy-functionalized implants have demonstrated their beneficial effects on osseointegration [26,27], leading to the filing of a national patent (BR 10 2021 025891 8).

Despite existing evidence on the individual effects of these biomolecules, there remains a gap in scientific knowledge regarding their combined application, especially in animal models with and without osteoporosis. Considering that the synergistic effect between an antiresorptive agent such as zoledronic acid and a nitric oxide donor like ruterpy may simultaneously preserve the mineral matrix and stimulate bone formation, this combination is highly relevant from a translational perspective.

Therefore, the present study aims to evaluate the impact of implant surface functionalization with zoledronic acid alone and in combination with ruterpy on peri-implant bone repair in healthy and osteoporotic rats. Through histological (H&E staining), micro-computed tomography (Micro-CT), and confocal microscopy analyses, we intend to comparatively investigate the effects of these interventions in two distinct systemic conditions in order to assess the potential of this strategy to promote osseointegration in both normal and osteoporotic contexts.

## 2. Materials and Methods

### 2.1. Animals

The present study complies with the ARRIVE guidelines [28] and national laws on animal use. All procedures conducted in this study were previously submitted for evaluation by the Animal Research Ethics Committee (CEUA) of the respective institution. Following the approval of the protocol (No. 0520-2022), the experimental phase began. A total of 66 adult female rats (Rattus norvegicus albinus, Wistar), aged between 3 and 4 months at the start of the experiment, were used. These animals were obtained from the central animal facility of the Araçatuba School of Dentistry, São Paulo State University “Júlio de Mesquita Filho” (UNESP). Throughout the experimental period, the rats were housed under controlled conditions, with a constant temperature of 22 ± 2 °C and a 12 h light/dark cycle. They had free access to water and a solid, balanced diet (Nuvilab, Curitiba, PR, Brazil). The animals were divided into two main groups: SHAM (healthy rats) and OVX (osteoporotic rats). Ninety days after bilateral ovariectomy or sham surgery, the animals were randomly assigned to one of three implant surface treatment subgroups within each main group, totaling six subgroups: SHAM CONV, SHAM ZOL, SHAM ZOL + TERPY, OVX CONV, OVX ZOL, and OVX ZOL + TERPY. The subgroups were defined as follows: CONV—animals receiving conventional implants without surface functionalization; ZOL—animals receiving implants coated with zoledronic acid by the dip-coating technique; ZOL + TERPY—animals receiving implants functionalized with both zoledronic acid and ruterpy using the dip-coating method. Each subgroup contained 11 rats, making a total of 66 animals, as shown in Table 1.

### 2.2. Experimental Model Design

Female Wistar rats (Rattus norvegicus albinus, Wistar, Philadelphia, PA, USA) subjected or not to bilateral ovariectomy were used as an experimental model to evaluate peri-implant bone repair under healthy and osteoporotic conditions. The tested drugs were zoledronic acid, administered either alone or in combination with ruterpy, both locally incorporated into the surface of implants placed in the proximal metaphyses of the tibiae. The analyses performed included qualitative histological evaluation using hematoxylin and eosin staining, micro-computed tomography (Micro-CT), and confocal microscopy. Several studies from our research group have already used the rat tibia implant installation model under different systemic conditions and with various implant surface treatments to observe peri-implant bone repair [26,27,28,29,30,31], obtaining interesting results.

**Figure 1:**
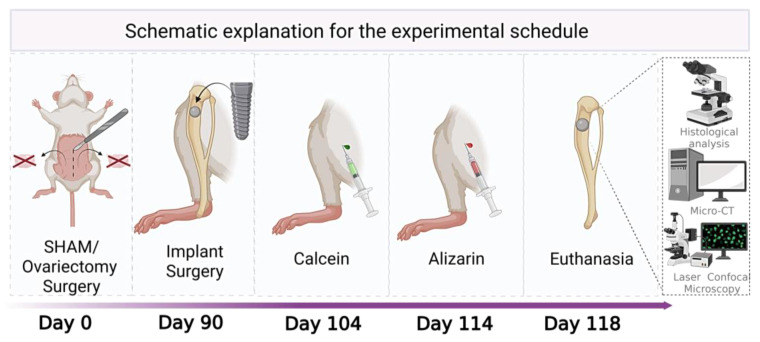
Illustrative diagram of the experimental schedule showing the sequence of experimental procedures and their respective execution periods.

On day 0, the rats underwent ovariectomy or sham surgery. After a 90-day period—sufficient for the development of osteoporosis in the OVX groups [32]—titanium implants were installed in the tibiae. Calcein fluorochrome was administered on day 104, followed by alizarin on day 114. Twenty-eight days after implantation (day 118), the animals were euthanized. One tibia from each animal was used for Micro-CT analysis and subsequently for confocal microscopy, allowing for correlation between the three-dimensional bone architecture and the mineralization dynamics during the healing process. The contralateral tibia was used for histological analysis. The assignment of the right or left tibia to each type of analysis was randomized using Excel 2016 software. The illustrative diagram of the experimental schedule can be found in Figure 1.

### 2.3. Implants Used in the Research

Titanium implants (grade IV, commercially pure) measuring 1.4 mm in diameter and 2.7 mm in length, manufactured with a surface modified by double-acid etching (Medens^®^, Itu, SP, Brazil), were selected for this study. Prior to surgical procedures, all implants were sterilized using gamma radiation.

To achieve surface functionalization, the implants were coated using the dip-coating technique, which consists of repeatedly immersing the implant in a solution containing the target compound to form uniform layers on its surface. During this procedure, the implants were stabilized vertically by coupling their internal hexagonal connectors to 100 µL pipette tips, which were then fixed upright in a mold made of alginate. This setup prevented any direct handling of the implants throughout the process, ensuring consistency and avoiding contamination or uneven deposition.

### 2.4. Surface Treatment of Implants

Depending on the experimental group, five or six coating layers were applied, based on preliminary findings indicating that a higher number of layers improved coating stability during implant placement. For implants assigned to the SHAM ZOL and OVX ZOL groups, five sequential immersions were performed in a zoledronic acid solution (Sigma-Aldrich, St. Loius, MO, USA), with a 24 h interval between each application. In the SHAM ZOL + TERPY and OVX ZOL + TERPY groups, six alternating layers were used—three initial immersions in the ruterpy solution (provided by the Faculty of Pharmaceutical Sciences, University of São Paulo), followed by three in the zoledronic acid solution, also spaced 24 h apart.

Solution concentrations were determined based on previous standardizations and literature data. The ruterpy working solution was prepared by diluting 10 mL of a concentrated stock (25 mg of ruterpy in 50 mL of Milli-Q^®^ water) in 40 mL of Milli-Q^®^ water and 50 mL of DMSO (dimethyl sulfoxide). For the zoledronic acid, 1 mg of the drug was dissolved in 1 mL of Milli-Q^®^ water and diluted further in 99 mL of DMSO, following established protocols [20,23,24]. All prepared solutions were kept at 18 °C in 100 mL beakers and were discarded after completion of the coating procedures.

### 2.5. In Vivo Procedures in the Osteoporotic Model

Following implant surface functionalization, in vivo experiments were conducted using the experimental healthy and osteoporosis model established in this study. The primary aim was to assess the synergistic effects of locally delivered drugs and broadly evaluate the early peri-implant bone tissue responses, including bone formation, resorption, and mineralization processes.

To simulate compromised bone conditions, the tibial metaphysis of ovariectomized rats was used as the implant site. This allowed investigation into the negative impact of osteoporosis and the potential regenerative effects of surface-modified implants treated with zoledronic acid or the combination of zoledronic acid and ruterpy via the dip-coating method.

### 2.6. Estrous Cycle Monitoring

To confirm that the rats selected for experimentation exhibited regular estrous cycles, each animal was housed individually. Daily, 1 to 2 drops of saline solution were introduced into the vaginal canal and then aspirated and placed on a glass slide for immediate microscopic evaluation, following the method established by Long and Evans (1922) [33]. This procedure enabled identification of the estrous cycle phase. Animals were only included after displaying two to three consecutive regular cycles. The same technique was repeated post-ovariectomy to validate surgical success and confirm cessation of cycling.

### 2.7. Ovariectomy Procedure

Rats underwent an eight-hour preoperative fast before anesthesia was administered intramuscularly using a combination of 5 mg/kg of xylazine hydrochloride (2% solution, Syntec, Tamboré, SP, Brazil) and 50 mg/kg of ketamine hydrochloride (Vetnil, Louveira, SP, Brazil). After shaving the surgical area, aseptic preparation was performed using 10% povidone–iodine (Riodeine Germicidal, Rioquímica, São José do Rio Preto, SP, Brazil). With the animal in lateral decubitus, a 1 cm incision was made on each flank. Subcutaneous tissues and peritoneum were carefully dissected to access the abdominal cavity. The ovaries and uterine horns were identified, ligated using 5-0 monofilament nylon sutures (Ethicon, Johnson, São José dos Campos, SP, Brazil), and surgically excised. Closure was performed in layers: the internal sutures used monofilament nylon (5-0), while external skin closure was completed with 4-0 silk sutures (Ethicon, Johnson, São José dos Campos, SP, Brazil). Immediately after surgery, 0.2 mL of benzathine penicillin G (Pentabiótico Veterinary Small Size, Fort Dodge Animal Health Ltd., Campinas, SP, Brazil) was administered intramuscularly as prophylaxis [34]. It is important to note that in the SHAM group rats, the same surgical steps were performed, except for the ligation and excision of the ovaries. The purpose of this sham surgery in the SHAM group was to mimic the same surgical stress experienced by the OVX group rats.

### 2.8. Implant Placement Surgery in the Tibia

Implant placement in the tibial metaphysis was performed 90 days after either bilateral ovariectomy or sham surgery (day 90). Prior to the procedure, the rats were fasted for eight hours and anesthetized intramuscularly with 50 mg/kg of ketamine hydrochloride (Vetnil, Louveira, SP, Brazil) and 5 mg/kg of xylazine hydrochloride (Syntec, Tamboré, SP, Brazil), following the same anesthesia protocol as in the ovariectomy surgery. After anesthesia induction, the medial areas of both tibias were shaved, and the skin was aseptically disinfected using 10% Povidone-Iodine Germicidal Solution (Rioquímica, São José do Rio Preto, SP, Brazil) along with topical povidone–iodine.

Using a sterile No. 15 scalpel blade (Feather Industries Ltd., Tokyo, Japan), a roughly 1 cm longitudinal incision was made over the metaphyseal region of each tibia. Soft tissues were carefully dissected and retracted with periosteal elevators to expose the bone surface for implant placement. A total of 132 titanium implants of commercially pure grade IV, measuring 1.4 mm in diameter and 2.7 mm in height, and sterilized via gamma irradiation, were installed (two implants per rat). Osteotomy was performed in both cortical layers using a 1.1 mm diameter spiral drill powered by an electric motor (BLM600^®^; Driller, São Paulo, SP, Brazil) operating at 1000 rpm. Throughout the drilling process, continuous irrigation with 0.9% isotonic sodium chloride solution (Physiological^®^, Laboratórios Biosintética Ltd., Ribeirão Preto, SP, Brazil) was maintained using a 20:1 reduction contra-angle handpiece (KaVo^®^, Kaltenbach & Voigt GmbH & Co., Biberach, Germany). The soft tissues were closed in multiple layers using interrupted sutures, with monofilament Nylon 5-0 (Ethicon, Johnson, São José dos Campos, SP, Brazil) applied to the deeper layers and Silk 4-0 multifilament (Ethicon, Johnson, São José dos Campos, SP, Brazil) used for the skin closure. Immediately following surgery, all animals received an intramuscular injection of 0.2 mL Benzathine Penicillin G (Pentabiótico Veterinary Small Size, Fort Dodge Animal Health Ltd., Campinas, SP, Brazil) as a prophylactic antibiotic.

### 2.9. Application of Fluorochromes

Fourteen days after implant placement (day 104), 20 mg/kg of calcein was administered intramuscularly as a fluorochrome marker. After 10 more days (day 114), 30 mg/kg of red alizarin was also administered intramuscularly as a second fluorochrome marker.

### 2.10. Euthanasia

Twenty-eight days after implant placement (day 118), the animals were euthanized via anesthetic overdose. Both tibiae were collected for further analysis, with one tibia from each animal designated for micro-computed tomography (Micro-CT) and subsequently used for confocal microscopy, while the contralateral tibia was reserved for histological evaluation.

### 2.11. Processing of Calcified and Decalcified Tissue Samples

The processing of calcified tissues was carried out for both micro-computed tomography (Micro-CT) and confocal microscopy analyses. For each animal, one tibia was designated for calcified tissue processing, while the contralateral tibia was processed as decalcified tissue. Importantly, the same tibia sample used for Micro-CT scanning was subsequently utilized for confocal microscopy analysis, which is why both techniques share the same processing protocol. This process began with fixation in 10% buffered formalin for 48 h (Reagentes Analíticos^®^, Dinâmica Odonto-Hospitalar Ltd., Catanduva, SP, Brazil), followed by washing under running water for 24 h and storage in 70% ethanol. Micro-CT scanning was then performed to morphologically and quantitatively evaluate peri-implant bone repair and formation, applying parameters based on the rodent bone microarchitecture guidelines by Bouxsein et al. [35], enabling comparison across experimental groups.

The contralateral tibia was processed as decalcified tissue, starting with fixation in 10% buffered formalin for 48 h, followed by a 24 h rinse under running water. Decalcification was achieved through approximately 16 successive changes of 10% EDTA solution over an eight-week period. After decalcification, samples were again washed for 24 h under running water, dehydrated in xylene, and embedded in paraffin. During the processing of the pieces for embedding in paraffin, the implants were removed by screwing them in an anticlockwise direction with a prosthetic wrench specifically designed for their connection. After being embedded in paraffin, they were subjected to microtomy and sectioned into 6 μm thick slices. These sections were mounted on slides and stained with hematoxylin and eosin for qualitative histological evaluation.

### 2.12. Micro-CT Three-Dimensional Analysis

Specimens were scanned using a SkyScan 1272 micro-CT system (Bruker MicroCT, Billerica, MA, USA) with an 8 μm slice thickness, operated at 90 kV and 111 μA, utilizing an Al 0.5 mm + Cu 0.038 filter. Images were acquired with a rotation step of 0.4° and pixel resolution of 2016 × 1344 μm over approximately 2 h. Raw projection data were reconstructed with NRecon software (v1.6.6.0) applying smoothing, ring artifact correction, and beam hardening adjustments. Transaxial images were evaluated in Data Viewer (v1.4.4).

For morphometric 3D analysis, CTAn software (v1.12.4.0) was used. The region of interest was defined by selecting 150 slices of the medullary bone adjacent to the im(plant, starting below the cortical bone. A grayscale threshold of 20–120 was applied to quantify bone parameters such as bone volume fraction (BV/TV), trabecular number (Tb.N), trabecular separation (Tb.Sp), trabecular thickness (Tb.Th), total porosity (Po.Tot), and bone-to-implant contact (I.S). Additionally, three-dimensional reconstructions of radiographic images were generated using CTvox^®^ software (SkyScan, Version 2.7) to visually demonstrate peri-implant bone formation across the various experimental groups.

### 2.13. Epifluorescence

The same specimens analyzed via Micro-CT were subsequently examined using confocal laser scanning microscopy to assess epifluorescence. Fluorochromes were administered at two specific postoperative intervals: calcein (20 mg/kg) on day 104 and alizarin (30 mg/kg) on day 114, with euthanasia occurring on day 118. Post-extraction, the tibiae were fixed in a 10% formaldehyde solution for 48 h, rinsed under running water for 24 h, and then preserved in 70% alcohol in preparation for microtomographic analysis. Following imaging, the samples underwent dehydration and were gradually embedded in a mixture of 100% alcohol and a thermo-polymerizing resin (methylmethacrylate-based acrylic resin) until only resin was used for complete immersion. Sections approximately 80 μm thick were obtained using the Exakt Cutting System, which automates both cutting and polishing processes.

Microscopy was performed using a Leica CTR 4000 CS SPE confocal system (Leica Microsystems, Heidelberg, Germany). Images were collected across various depths in the implant-adjacent region and processed using z-stack imaging to optimize visual clarity. Each image had a resolution of 512 × 512 pixels and covered an area of 1 × 1 mm^2^. Scanning was performed in 2 μm increments over 2.5 min, yielding 28 optical slices for every 56 μm scanned. Fluorescent detection employed BP 530/30 nm and 590 LP filters along with a dual-dichroic beam splitter (488/568 nm). Calcein and alizarin signals were detected using photomultiplier settings of 534 and 357, respectively. These corresponded to filters that visualized calcein in blue and alizarin in green.

The resulting image stacks were reconstructed using proprietary Leica software LAS X. In the peri-implant region, overlapping fluorochrome signals indicated zones of calcium deposition, with calcein marking earlier mineralization and alizarin reflecting more recent bone formation. Final images were exported in TIFF format and processed in ImageJ (version 1.52v, NIH, Bethesda, MD, USA). Color thresholding was applied to isolate the fluorochromes based on hue, saturation, and brightness. Calcein-labeled areas (green) were measured first, followed by alizarin-labeled regions (red), enabling assessment of bone remodeling dynamics. Since calcein was administered 10 days prior to alizarin, the spatial distance between fluorochrome bands was used to calculate the mineral apposition rate (MAR), representing the rate of new bone deposition over time.

### 2.14. Qualitative Histological Evaluation

Following decalcification with 10% EDTA, tibial samples were rinsed for 24 h under running water, dehydrated through increasing alcohol concentrations, cleared in xylene, and embedded in paraffin. Sections of 6 μm thickness were cut using a Leica RM2125 RTS microtome. These slices were then mounted on slides and stained with hematoxylin for 8 min and 1% eosin for 30 s. Coverslips were applied, and the samples were examined under a Leica DM 750 optical microscope at 4×, 10×, and 20× magnifications. Subsequently, the slides were scanned using the Motic Easy Scan device, and the peri-implant region was digitally captured using the Motic DSAssistant (4K) software (version 1.0.7.61b) at magnifications of 4×, 10×, and 20×. The images obtained were saved in JPG format.

### 2.15. Statistical Analysis

The numerical data obtained from Micro-CT and confocal microscopy (MAR parameter) analysis were analyzed using GraphPad Prism 7.03 software. Normality was confirmed with the Shapiro–Wilk test, followed by two-way ANOVA and Tukey’s post hoc test when needed. Statistical significance was set at *p* < 0.05. Sample size calculation was based on the BV/TV parameter, referencing Gomes-Ferreira et al. [30]. Using OpenEpi’s power calculator (accessed 9 November 2022), group means of 33.37 and 43.54 with standard deviations of 8.53 and 5.77, a 5% significance level, and 95% power determined that 11 animals per group were needed. Power was set at 0.8 with an alpha of 0.05.

## 3. Results

### 3.1. Confirmation of the Efficacy of Bilateral Ovariectomy Surgery

Eight days following bilateral ovariectomy, monitoring of the animals’ estrous cycle indicated a persistent diestrus phase, confirming the effectiveness of the surgical induction. Ninety days post-ovariectomy, implants were placed in the tibial metaphysis, at which point osteoporotic conditions were considered fully developed [31].

### 3.2. Qualitative Histological Results

**Figure 2:**
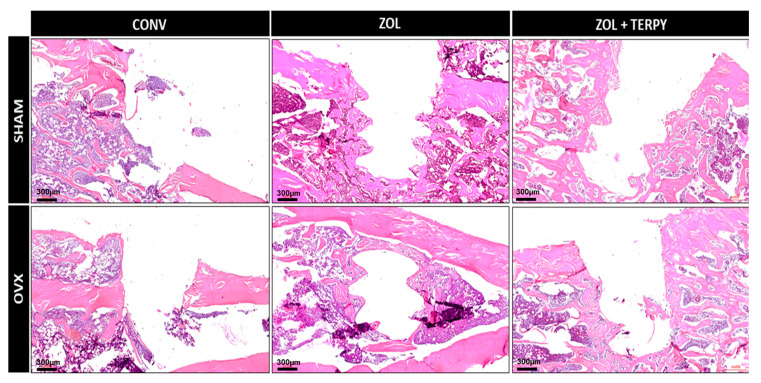
Hematoxylin and eosin staining of SHAM CONV, SHAM ZOL, SHAM ZOL + TERPY, OVX CONV, OVX ZOL, and OVX ZOL + TERPY groups. Slides are shown at 4× magnification. Scale bar: 300 µm.

**Figure 3:**
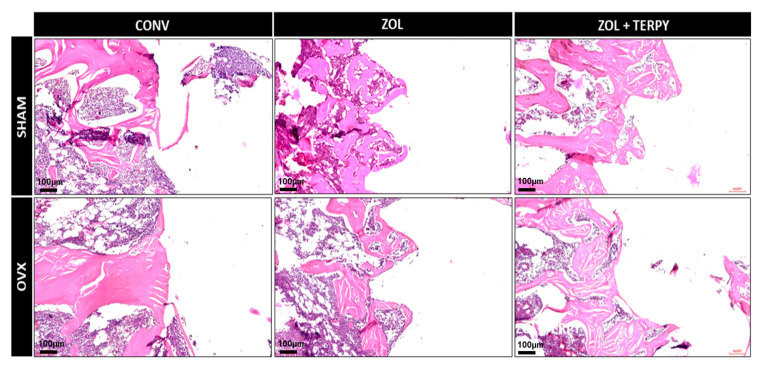
Hematoxylin and eosin staining of SHAM CONV, SHAM ZOL, SHAM ZOL + TERPY, OVX CONV, OVX ZOL, and OVX ZOL + TERPY groups. Slides are shown at 10× magnification. Scale bar: 100 µm.

**Figure 4:**
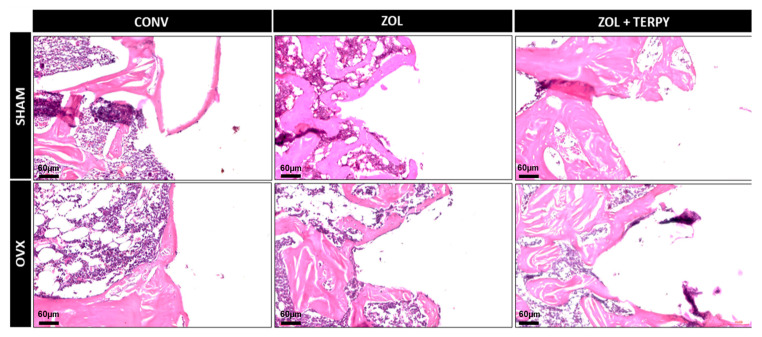
Hematoxylin and eosin staining of SHAM CONV, SHAM ZOL, SHAM ZOL + TERPY, OVX CONV, OVX ZOL, and OVX ZOL + TERPY groups. Slides are shown at 20× magnification. Scale bar: 60 µm.

Figure 2, Figure 3 and Figure 4 present histological images at different magnifications in order to progressively illustrate the general morphology and the finer structural details of the peri-implant region.

#### 3.2.1. SHAM CONV and OVX CONV Groups

At 28 days postoperatively, the peri-implant morphological appearance was similar for both groups: a predominance of disorganized connective tissue was observed in the peripheral peri-implant region, surrounded only by a few islands of mature bone tissue. It was also not possible to observe the morphological definition of the implant threads in any of the groups, indicating that bone repair did not occur in close contact with the implant, even in the SHAM CONV group (Figure 2, Figure 3 and Figure 4).

#### 3.2.2. SHAM ZOL and OVX ZOL Groups

At 28 days postoperatively, significant peri-implant morphological changes were observed in the SHAM ZOL and OVX ZOL groups compared to the control groups: there was a marked increase in the volume of mature bone tissue in the peri-implant region, with little interposed connective tissue in the SHAM ZOL group, and no clear predominance between bone and connective tissue in the OVX ZOL group. Furthermore, bone tissue formation was also observed in close contact with the implant surface, resulting in a clear morphological definition of the implant threads, especially in the SHAM ZOL group (Figure 2, Figure 3 and Figure 4).

#### 3.2.3. SHAM ZOL + TERPY and OVX ZOL + TERPY Groups

At 28 days postoperatively, an even greater volume and organization of bone tissue in the peri-implant region were observed in the SHAM ZOL + TERPY and OVX ZOL + TERPY groups compared to the SHAM ZOL and OVX ZOL groups. Qualitatively, the SHAM ZOL + TERPY group appeared to show the best bone matrix maturation and organization, with the least amount of interposed connective tissue. This group also exhibited the clearest definition of the peri-implant threads among all experimental groups. (Figure 2, Figure 3 and Figure 4).

### 3.3. Three-Dimensional Microtomographic (Micro-CT) Results

**Figure 5:**
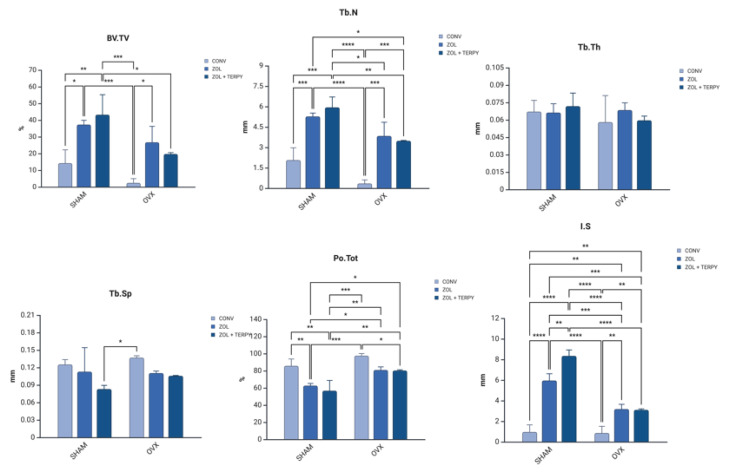
Micro-CT results: SHAM CONV, SHAM ZOL, SHAM ZOL + TERPY, OVX CONV, OVX ZOL, and OVX ZOL + TERPY groups. Results are presented as the mean ± SD for each group. Statistically significant difference: * (<0.05); ** (<0.005); *** (<0.0005); **** (<0.00005).

The results are presented in Table 2 and Figure 5.

### 3.4. Three-Dimensional Microtomographic Reconstructions (CTvox^®^ Software)

**Figure 6:**
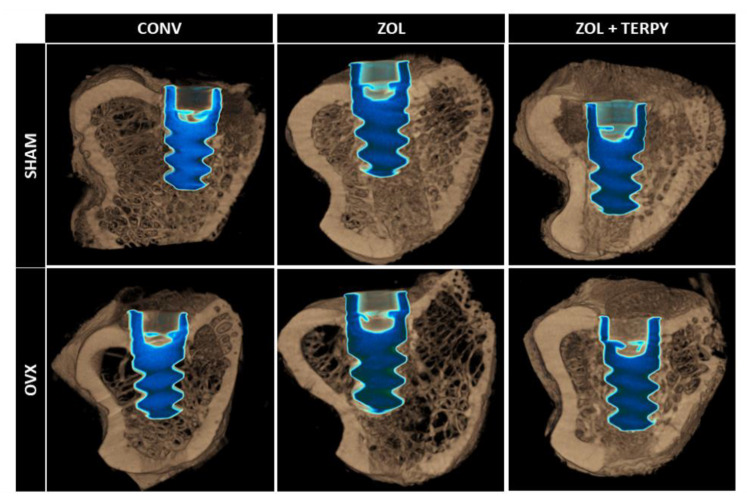
Three-dimensional Micro-CT reconstructions of the SHAM CONV, SHAM ZOL, SHAM ZOL + TERPY, OVX CONV, OVX ZOL, and OVX ZOL + TERPY groups generated using CTvox^®^ software (SkyScan, version 2.7). The green color corresponds to the deposition of the fluorochrome calcein, while the red color corresponds to the deposition of the fluorochrome alizarin.

Visually and qualitatively, a seemingly higher density of peri-implant bone tissue can be observed in the groups that received implants with surface functionalization compared to the groups without functionalization. Additionally, a lower bone density is noted in all OVX groups compared to the SHAM groups, demonstrating the effect of bilateral ovariectomy on the quantity and quality of bone tissue, as illustrated in Figure 6.

### 3.5. Epifluorescence Results

#### 3.5.1. Bone Dynamics

**Figure 7:**
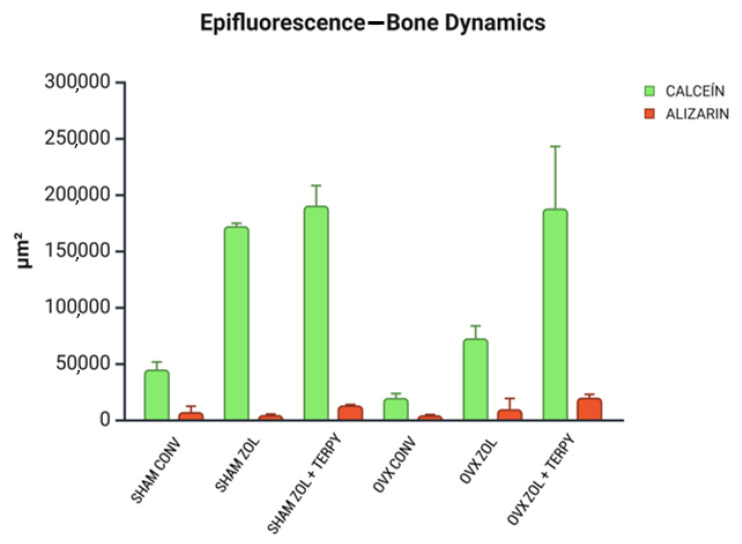
The confocal microscopy results analyzed bone dynamics: for the fluorochromes of calcein and alizarin precipitations at 14 and 24 days after implantation, respectively. The calcein is evidence of an older bone, while the alizarin indicates a new bone. Results are presented as the mean ± SD for each group.

**Figure 8:**
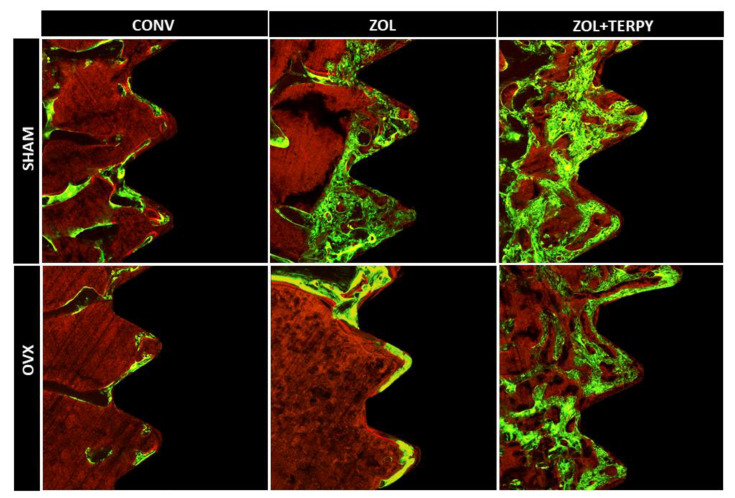
Bone dynamics. A representative image of bone dynamics formed by overlapping the fluorochromes of calcein and alizarin in the peri-implant bone of the tibia in each group for the SHAM CONV, SHAM ZOL, SHAM ZOL + TERPY, OVX CONV, OVX ZOL, and OVX ZOL + TERPY groups after 28 days of implantation using ImageJ software (version 1.52v, National Institutes of Health, Bethesda, MD, USA). The blue color corresponds to the titanium implants, while the brown color corresponds to the bone tissue.

The bone mineral precipitation from confocal analysis showed that ZOL and ZOL + TERPY implant surface functionalizations have therapeutic effects in peri-implant bone dynamics. The results of fluorochrome precipitation are shown, respectively, for calcein and alizarin: SHAM ZOL + TERPY (190.260 ± 14.438 and 12.889 ± 0.553), OVX ZOL + TERPY (187.717 ± 31.763 and 19.814 ± 1.608) (SHAM ZOL—172.069 ± 1.747 and 4.296 ± 0.592), (OVX ZOL—72.483 ± 8.857 and 9.499 ± 7.743), (SHAM CONV—44.775 ± 5.365 and 7.007 ± 4.158), and (OVX CONV—19.554 ± 3.098 and 4.142 ± 0.417). The results showed an acceleration of the peri-implant repair process in the groups that received functionalized implants, especially in the groups functionalized with zoledronic acid in combination with ruterpy (SHAM ZOL + TERPY and OVX ZOL + TERPY). This conclusion can be drawn due to the notably greater calcein deposition (which indicates older bone) in these groups compared to the control groups (Figure 7). A descriptive image of bone dynamics formed by overlapping the fluorochromes of calcein and alizarin in the peri-implant bone is shown in Figure 8.

#### 3.5.2. Daily Mineral Apposition Rate (MAR)

**Figure 9:**
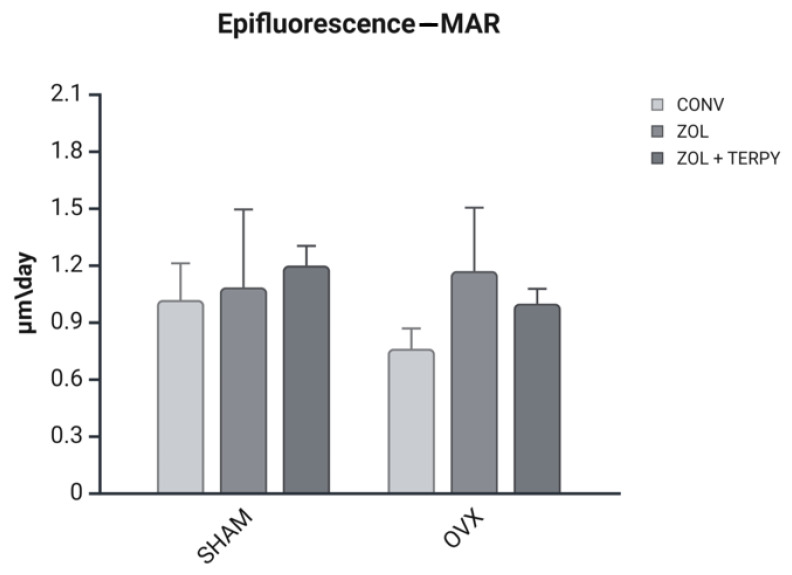
Mineral apposition rate (MAR). The MAR was measured to evaluate the rate of bone formation in response to systemic conditions (SHAM/OVX) and implant surface treatment (CONV/ZOL/ZOL + TERPY). Data are expressed as the distance between two fluorochrome labels (calcein and alizarin red) divided by the time interval between the injections. Results are presented as the mean ± SD for each group.

The MAR values are reported in micrometers per day (µm/day) in Figure 9. There were no statistical differences between the groups.

## 4. Discussion

Osteoporosis is a systemic condition that significantly compromises the quality and quantity of bone tissue, hindering the bone repair process and the osseointegration of dental implants. Several studies have shown that in experimental models of osteoporosis, such as ovariectomized rats, there is a reduction in peri-implant bone formation [27,31]. Faced with this challenge, therapeutic strategies have been proposed, including the functionalization of implant surfaces with drugs that modulate bone metabolism.

Zoledronic acid, a powerful anti-resorptive bisphosphonate, has already shown positive results in preserving the bone matrix by inhibiting osteoclastic activity. However, as it acts mainly to inhibit resorption, its effect on active bone formation is limited [36]. On the other hand, ruterpy, a ruthenium complex that releases nitric oxide, has osteoinductive and angiogenic properties, promoting active bone formation [37,38,39]. Although the functionalization of implant surfaces with zoledronic acid has been previously investigated in animal models [19,20,21,22,23,24,25,26], no study to date has combined this agent with another drug with a complementary mechanism of action, such as ruterpy. Thus, the main aim of this study is to test the hypothesis that the synergistic combination of an anti-resorptive agent and an osteoforming agent, applied directly to the implant surface, can enhance peri-implant bone repair in osteoporotic rats. In addition, we sought to assess whether this approach would also bring benefits under physiological conditions (SHAM rats). Our findings show that both zoledronic acid and its association with ruterpy were effective in promoting bone repair, with more favorable histological, microtomographic, and bone dynamics evidence compared to the control groups.

The current study also builds upon previous findings by demonstrating that implant functionalization with zoledronic acid (ZOL), especially in combination with ruterpy (TERPY), improves and accelerates peri-implant repair in both healthy and osteoporotic rats—an effect previously shown only in osteoporotic models [26]. Additionally, confocal microscopy analysis, not performed in that earlier study, confirmed the acceleration of bone repair. The study further expands on results from another study [27], which reported the isolated effect of ruterpy by showing the enhanced action of ruterpy combined with ZOL under both physiological and osteoporotic conditions.

Histological analysis revealed greater formation and organization of bone tissue in the ZOL groups compared to the CONV groups. This trend was even more pronounced in the ZOL + TERPY groups, which showed thicker trabeculae and better filling of the bone–implant interface both in healthy and osteoporotic animals. These results were corroborated by the microtomographic data: we observed a significant increase in the BV/TV and Tb.N parameters, as well as a reduction in Po.Tot and Tb.Sp in the ZOL + TERPY groups, indicating greater bone volume and trabecular connectivity and less porosity. Confocal analysis also reinforces these findings, since the greater deposition of calcein in the ZOL + TERPY groups indicates accelerated mineral apposition and more intense osteoblastic activity during the 14-day postoperative period.

Zoledronic acid, a bisphosphonate from the nitrogen class, acts by inhibiting farnesyl–pyrophosphate synthase in the mevalonate pathway in osteoclasts, leading to apoptosis of these cells and a significant reduction in bone resorption [40]. This anti-resorptive effect promotes a more stable environment for the recruitment and differentiation of osteoblasts, favoring peri-implant bone repair. Our microtomographic and confocal data show this effect, since even ZOL alone was significantly superior to the CONV group.

Histological analysis revealed greater formation and organization of bone tissue in the ZOL groups compared to the CONV groups. This trend was even more pronounced in the ZOL + TERPY groups, which showed thicker trabeculae and better filling of the bone–implant interface both in healthy and osteoporotic animals. These results were corroborated by microtomographic data.

Ruterpy, in turn, is a ruthenium-based organometallic complex capable of controlled release of nitric oxide (NO), a key molecule in the regulation of osteoblastic and osteoclastic activity. NO acts to promote angiogenesis and the differentiation of mesenchymal stem cells, as well as increase the expression of osteoinductive genes such as Runx2 and osteocalcin [41,42,43]. The association of ruterpy with ZOL suggests a synergistic effect between inhibiting resorption and stimulating bone formation, which translates into higher parameters in most of the analyses carried out. These findings reinforce previous studies by our group, which have shown similar beneficial effects on peri-implant bone repair with the isolated or combined use of these medications [26,27]. Histological analysis revealed greater formation and organization of bone tissue in the ZOL groups compared to the CONV groups. It is important to note that in the osteoporotic groups (OVX), implant functionalization proved to be particularly effective. The literature already shows that osteoporosis significantly impairs peri-implant bone repair, with a decrease in bone mineral density, trabecular rarefaction, increased porosity, and delayed bone formation. These effects are mainly due to the imbalance between bone formation and resorption, which is driven by greater osteoclastic activity and osteoblastic dysfunction [2,3,4]. Our findings corroborate these studies, since the OVX CONV groups showed drastically lower values in histological and microtomographic parameters. However, functionalization with ZOL and especially ZOL + TERPY was able to attenuate these deleterious effects, promoting bone repair patterns closer to those observed in healthy animals.

Therefore, the results of this study point to the promising therapeutic potential of the combination of zoledronic acid and ruterpy as a local approach for optimizing peri-implant repair, especially in osteoporotic contexts. Functionalizing the surface of implants with these bioactive molecules may represent a viable strategy for overcoming the biological limitations imposed by osteoporosis and improving clinical outcomes in patients with low bone mineral density.

However, the limitations of this study are mainly based on the lack of a more robust physicochemical characterization of the surface of the ZOL and ZOL + TERPY implants. The thickness and uniformity of the drug layer, the drug release profile, and the resistance of the functionalization layer to mechanical abrasion were not investigated, along with the presence of NO or VEGF in the peri-implant bone tissue.

## 5. Conclusions

The functionalization of implant surfaces with the combination of zoledronic acid and ruterpy was able to promote, in both healthy and osteoporotic rats, not only an acceleration of the peri-implant repair process but also better organization, greater quality, and increased quantity of newly formed bone tissue compared to the group functionalized with zoledronic acid alone and, especially, in relation to the control group.

## 6. Patents

BR 10 2021 025891 8 process—“Funcionalização da superfície de implantes com molécula doadora de óxido nítrico através da técnica layer by layer como estratégia para a melhora do processo de reparo periimplantar em ratas” (870210118781 petition).

## Figures and Tables

**Table 1:** Description of the experimental groups according to systemic condition and implant surface functionalization.

Group Name	*N*	Description of the Group
SHAM CONV	11	Implants without surface functionalization installed in healthy rats
SHAM ZOL	11	Implants functionalized with zoledronic acid installed in healthy rats
SHAM ZOL + TERPY	11	Implants functionalized with zoledronic acid + ruterpy installed in healthy rats
OVX CONV	11	Implants without surface functionalization installed in osteoporotic rats
OVX ZOL	11	Implants functionalized with zoledronic acid installed in osteoporotic rats
OVX ZOL + TERPY	11	Implants functionalized with zoledronic acid + ruterpy installed in osteoporotic rats

**Table 2:** Micro-CT results of SHAM CONV, SHAM ZOL, SHAM ZOL + TERPY, OVX CONV, OVX ZOL, and OVX ZOL + TERPY groups. Results are presented as mean ± standard deviation (SD).

	SHAM CONV	SHAM ZOL	SHAM ZOL + TERPY	OVX CONV	OVX ZOL	OVX ZOL + TERPY	Statistical Difference
BV.TV (%)	14.24 ± 7.98	37.32 ± 2.60	43.26 ± 12.00	2.435 ± 2.54	26.72 ± 9.58	19.71 ± 0.84	SHAM CONV × SHAM ZOL = 0.01 SHAM CONV × SHAM ZOL + TERPY = 0.0037 OVX CONV × SHAM ZOL = 0.0007819 OVX CONV × OVX ZOL = 0.01400 OVX CONV × SHAM ZOL + TERPY = 0.0001822 SHAM ZOL + TERPY × OVX ZOL + TERPY = 0.01731
Tb.Th (mm)	0.067 ± 0.009	0.066 ± 0.007	0.071 ± 0.011	0.058 ± 0.022	0.068 ± 0.006	0.059 ± 0.003	no
Tb.N (per/mm)	2.067 ± 0.905	5.278 ± 0.247	5.941 ± 0.765	0.351 ± 0.260	3.842 ± 1.007	3.483 ± 0.029	SHAM CONV × SHAM ZOL = 0.0006514 SHAM CONV × SHAM ZOL + TERPY = 0.0001 OVX CONV × SHAM ZOL ≤.0001 OVX CONV × OVX ZOL = 0.0003016 OVX CONV × SHAM ZOL + TERPY ≤0.0001 OVX CONV × OVX ZOL + TERPY = 0.0008137 SHAM ZOL × OVX ZOL + TERPY = 0.04907 OVX ZOL × SHAM ZOL + TERPY = 0.01870 SHAM ZOL + TERPY × OVX ZOL + TERPY = 0.006051
Tb.Sp (mm)	0.125 ± 0.008	0.112 ± 0.041	0.083 ± 0.006	0.136 ± 0.003	0.110 ± 0.003	0.105 ± 0.000	OVX CONV × SHAM ZOL + TERPY = 0.02496
Po.Tot (%)	85.75 ± 7.989	62.68 ± 2.607	56.74 ± 12.000	97.57 ± 2.543	80.91 ± 3.660	80.29 ± 0.849	SHAM CONV × SHAM ZOL = 0.007166 SHAM CONV × SHAM ZOL + TERPY = 0.0011 OVX CONV × SHAM ZOL = 0.0002067 OVX CONV × SHAM ZOL + TERPY ≤0.0001 OVX CONV × OVX ZOL + TERPY = 0.04829 SHAM ZOL × OVX ZOL = 0.03534 SHAM ZOL × OVX ZOL + TERPY = 0.04333 OVX ZOL × SHAM ZOL + TERPY = 0.005048 SHAM ZOL + TERPY × OVX ZOL + TERPY = 0.0061510
IS (mm)	0.963 ± 0.693	5.959 ± 0.657	8.347 ± 0.579	0.861 ± 0.650	3.184 ± 0.465	3.108 ± 0.093	SHAM CONV × SHAM ZOL = <0.0001 SHAM CONV × OVX ZOL = 0.004228 SHAM CONV × SHAM ZOL + TERPY ≤0.000 SHAM CONV × OVX ZOL + TERPY = 0.005554 OVX CONV × SHAM ZOL ≤0.0001 OVX CONV × OVX ZOL = 0.002953 OVX CONV × SHAM ZOL + TERPY ≤0.0001 OVX CONV × OVX ZOL + TERPY = 0.003866 SHAM ZOL × OVX ZOL = 0.0006402 SHAM ZOL × SHAM ZOL + TERPY = 0.002356 SHAM ZOL × OVX ZOL + TERPY = 0.0005000 OVX ZOL × SHAM ZOL + TERPY ≤0.0001 SHAM ZOL + TERPY × OVX ZOL + TERPY ≤0.0001

## Data Availability

Data are contained within this article.
